# Identification of trombiculid mites (Acari: Trombiculidae) on rodents from Chiloé Island and molecular evidence of infection with *Orientia* species

**DOI:** 10.1371/journal.pntd.0007619

**Published:** 2020-01-23

**Authors:** Gerardo Acosta-Jamett, Constanza Martínez-Valdebenito, Esperanza Beltrami, María Carolina Silva-de La Fuente, Ju Jiang, Allen L. Richards, Thomas Weitzel, Katia Abarca

**Affiliations:** 1 Instituto de Medicina Preventiva Veterinaria, Facultad de Ciencias Veterinarias, Universidad Austral de Chile, Valdivia, Chile; 2 Programa de Investigación Aplicada en Fauna Silvestre, Facultad de Ciencias Veterinarias, Universidad Austral de Chile, Valdivia, Chile; 3 Departamento de Enfermedades Infecciosas e Inmunología Pediátricas, Escuela de Medicina, Pontificia Universidad Católica de Chile, Santiago, Chile; 4 Laboratorio de Infectología y Virología Molecular, Red Salud UC–Christus, Santiago, Chile; 5 Escuela de Graduados, Facultad de Ciencias Veterinarias, Universidad Austral de Chile, Valdivia, Chile; 6 Departamento de Ciencias Animal, Facultad de Ciencias Veterinarias, Universidad de Concepción, Concepción, Chile; 7 Programa de Doctorado en Ciencias Veterinarias, Facultad de Ciencias Veterinarias, Universidad de Concepción, Chillán, Chile; 8 Facultad de Medicina Veterinaria, Universidad San Sebastián, Concepción, Chile; 9 Viral and Rickettsial Diseases Department, Naval Medical Research Center, Silver Spring, MD, United States of America; 10 Department of Preventive Medicine and Biostatistics, Uniformed Services University of the Health Sciences, Bethesda, MD, United States of America; 11 Laboratorio Clínico, Clínica Alemana de Santiago, Facultad de Medicina Clínica Alemana, Universidad del Desarrollo, Santiago, Chile; 12 Hantavirus and Zoonoses Program, Instituto de Ciencias e Innovación en Medicina (ICIM), Facultad de Medicina Clínica Alemana, Universidad del Desarrollo, Santiago, Chile; 13 Millennium Institute on Immunology and Immunotherapy, Escuela de Medicina, Pontificia Universidad Católica de Chile, Santiago, Chile; AUSTRALIA

## Abstract

**Background:**

Scrub typhus is an emerging vector-borne zoonosis, caused by *Orientia* spp. and transmitted by larvae of trombiculid mites, called chiggers. It mainly occurs within a region of the Asia-Pacific called the tsutsugamushi triangle, where rodents are known as the most relevant hosts for the trombiculid vector. However, the reservoir(s) and vector(s) of the scrub typhus outside Asia-Pacific are unknown. The disease has recently been discovered on and is considered endemic for Chiloé Island in southern Chile. The aim of the present work was to detect and determine the prevalence of chiggers on different rodent species captured in probable sites for the transmission of orientiae responsible for scrub typhus on Chiloé Island in southern Chile and to molecularly examine collected chiggers for the presence of *Orientia* DNA.

**Methodology/Principal findings:**

During the austral summer 2018, rodents were live-trapped in six sites and examined for chigger infestation. All study sites were rural areas on Chiloé Island, previously identified as probable localities where human cases acquired the scrub typhus. During a total of 4,713 trap-nights, 244 rodents of seven species were captured: the most abundant was *Abrothrix olivacea*. Chiggers were detected on all seven rodent species with a 55% prevalence rate. Chiggers showed low host specificity and varied according to site specific host abundance. Three genera of trombiculids were identified. *Herpetacarus* was the most abundant genus (93%), prevalent in five of the six sites. Infestation rates showed site specific differences, which were statistically significant using a GLM model with binomial errors. Molecular analyses proved that 21 of 133 (15.8%) mite pools were positive for *Orientia* species, all of them belonged to the genus *Herpetacarus*.

**Conclusions/Significance:**

This study firstly reports the presence of different rodent-associated chigger mites positive for *Orientia* sp., in a region endemic for scrub typhus in southern Chile. *Herpetacarus* and two other genera of mites were found with high infestation rates of rodents in sites previously identified as probable exposure of scrub typhus cases. A substantial percentage of mite pools were positive for *Orientia* DNA, suggesting that chigger mites serve as vectors and reservoirs of this emerging zoonosis in South America.

## Author summary

Scrub typhus is a chigger-transmitted zoonotic infection caused by *Orientia* species, which is endemic to the tsutsugamushi triangle in Asia-Pacific region. Recently, a focus of scrub typhus in South America has been confirmed on Chiloé Island in southern Chile. However, the vectors of scrub typhus in this region remain unknown. We undertook a survey to study the presence of chiggers on different rodent species in areas identified as probable sites of exposure to scrub typhus on Chiloé Island. The study showed that 55% of rodents were infested by trombiculids. Three mite genera were identified, of which *Herpetacarus* was the most abundant. Chiggers showed low host specificity, but spatial differences. Using molecular techniques, the trombiculid mites were found to be infected with *Orientia* species. These findings suggest that chigger mites play a role in the life cycle and transmission of this emerging infectious disease in Chile.

## Introduction

Scrub typhus is a zoonotic disease caused by bacteria of the genus *Orientia*, which causes significant morbidity and mortality [1]. The disease was previously thought to be restricted to a certain region, known as the tsutsugamushi triangle, in Asia-Pacific, but recent cases from the Arabian Peninsula and southern Chile have called this paradigm into question [2–4]. This recent expansion of the endemic region is supported by serological data and studies in animals mainly from Africa [5].

In the Asia-Pacific area, the orientia infection is transmitted by larvae of trombiculid mites known as chiggers, which are also the reservoir of the orientiae through transovarial and transstadial transmission. Small vertebrates, usually rodents, serve as main hosts of the chiggers and are a critical part of the epidemiology of scrub typhus in the Asia-Australia-Pacific region [6]. Cases mainly occur in rural areas, where populations of infected trombiculid mites are present with a patchy distribution (mite islands) [1]. Surveillance of chiggers has been used as a proxy for the spatial risk of scrub typhus in humans [7, 8]. In Asia-Pacific, bacteria is transmitted by different species of *Leptotrombidium* [9], the transmission by other genera in Korea (*Euschoengastia*, *Neotrombicula*), Japan (*Schoengastia*), and India (*Schoengastiella*) has been suggested, but remains controversial [10, 11]. A key factor to understand the distribution and emergence of scrub typhus in these regions is the knowledge of the local chigger fauna, their rodent hosts, and their interaction with environmental and climatic factors [eg. 12, 13].

Recently, the first endemic focus of scrub typhus in South America has been confirmed on Chiloé Island in southern Chile [2, 3]. Still, the vectors and reservoirs of scrub typhus in regions outside the tsutsugamushi triangle remain unknown. In Chile, 18 species of trombiculid mites have been reported so far, mostly from reptiles [14–16]. Still, none of them belong to the genus *Leptotrombidium* or other genera associated with orientia, and up to now, no studies regarding the rodent-associated chigger fauna have been performed in scrub typhus endemic regions in southern Chile. The aim of the present work was to study the presence, prevalence, and distribution of chiggers and analyse their infestation pattern on different rodent species captured in sites, which were identified in previous studies as probable hot spots of scrub typhus on Chiloé Island. To study them as possible vectors of scrub typhus in Chile, chigger mite samples were molecularly examined for the presence of *Orientia* DNA.

## Methods

### Study sites

Chiloé Island belongs to Los Lagos region in southern Chile and is the second largest island in Chile with an area of 8,394 km^2^. The climate is oceanic temperate, with mean annual precipitations of 2,090 mm and an average annual temperature of 12°C [17]. The original vegetation is Valdivian temperate rain forest, which has been highly fragmented by clearing for livestock rising and timber extraction [18], and the current matrix involves mainly pastures and secondary scrublands [19].

**Fig 1:**
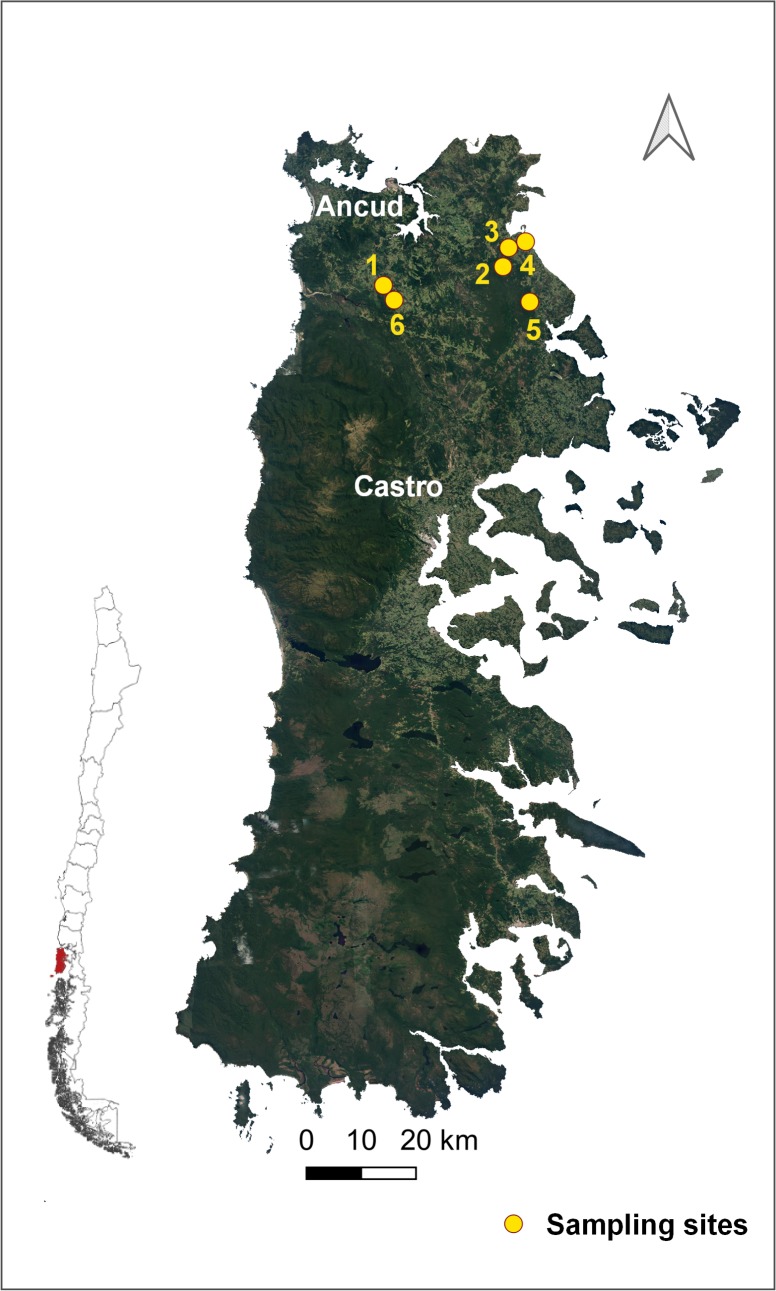
Study area in rural localities of the north-eastern area of Chiloé Island, Los Lagos Region, Chile. (Map made in QGIS Geographic Information System. Open Source Geospatial Foundation Project. http://qgis.osgeo.org. Shapes downloaded from an open source from the Biblioteca del Congreso Nacional, Available at https://www.bcn.cl/siit/mapas_vectoriales/index_html).

During the austral summer months of January and February 2018, small mammals were live-trapped at six sites in the northern part of Chiloé Island (Fig 1). Study sites had been identified as possible areas of exposure of scrub typhus cases [3]. All sites consisted of partially cleared forest due to timber activities, with remaining native lower vegetation. Localities were geo-referenced by GPS and located into a digitalized map using Arc Gis 10.1 (Esri, New York).

### Trapping and parasite sampling

**Fig 2:**
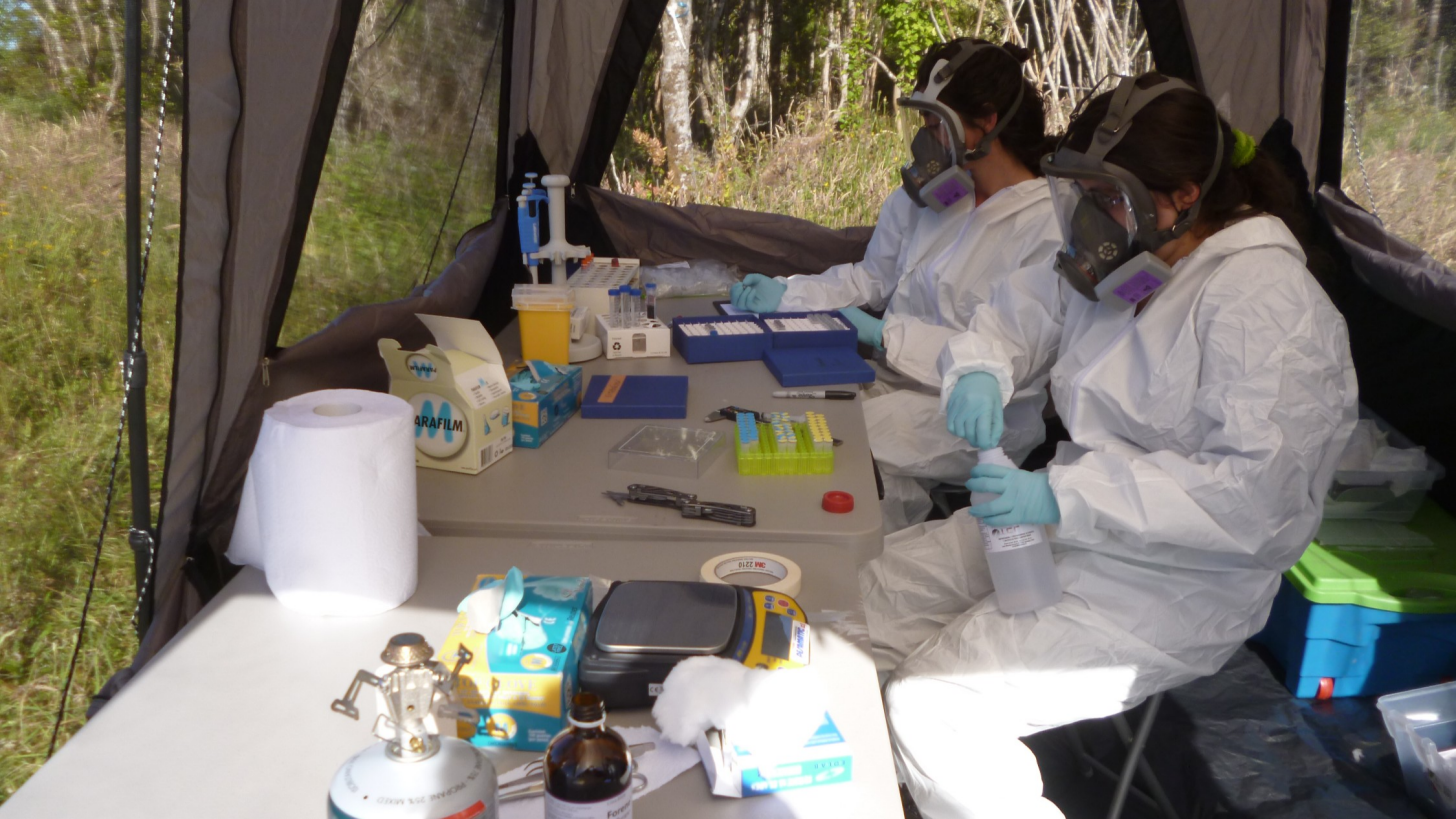
Central processing tent installed at each sampling site. Biosafety conditions during rodent handling were adapted to the risk of hantavirus cardiopulmonary syndrome.

A total of 148 to 175 Sherman-like traps (300 x 100 x 110 mm) were set up during four to five consecutive nights at each site. Trapping was operated for a total of 4,713 trap-nights and ranged from 668 to 895 trap-nights per site (average 785.5). Traps were situated ≥5 meters apart and placed under scrub, fallen logs, understory or burrows, baited with oat flakes and vanilla essence, and conditioned both inside and outside with vegetal material to protect animals from cold and rain. Traps were activated at sunset, checked early the next morning, and closed during the day to avoid capturing non-target species. Captured rodents were moved to a central processing tent installed at the sampling site (Fig 2), where they were chemically immobilized using an induction chamber containing cotton embedded with isoflurane (1 ml of isoflurane per 500 ml of chamber volume). After anaesthesia, male and juvenile female rodents were euthanized by cervical dislocation; adult females were marked by haircut and released at the respective capture points. Rodents species were identified by morphological criteria following Iriarte [20]. Each rodent was thoroughly examined for the presence of ectoparasites by brushing the body with a fine comb over a plate covered with water. In adult females with high mite loads, we also conducted a careful scraping of the perianal zone. Chigger mites were collected from the water surface and placed in tubes with 95% ethanol. The skin of euthanized rodents, including ears and perianal zones, was dissected, stored in falcon tubes with 95% ethanol, and later revised for additional chiggers in the entomological laboratory.

### Identification of mites

Taxonomic analyses of trombiculid mites were performed at the Laboratorio de Parásitos y Enfermedades de Fauna Silvestre, Universidad de Concepción in Chillán, Chile. Firstly, mite specimens of the individual rodents were pooled by their macroscopic appearance (morphotypes). Preserved rodent samples of skin and ears were checked for additional mites, which were added to the respective pools. One individual of each pool was cleared in Nesbitt's solution and mounted in Berlese's medium [21]. Specimens were then identified under an optical microscope (Leica DM 1000 LED) at 400x magnification, following nomenclature and methodology of Brennan & Goff [22].

Southern Chile is endemic for Andes virus, a rodent-borne virus causing hantavirus cardiopulmonary syndrome [23]. Therefore, the handling of captured animals strictly followed the guidelines of the Centers for Disease Control and Prevention (CDC) [24] and the American Society of Mammalogists [25] for such regions. Personal protective equipment included masks with HEPA filters as well as disposable gowns and gloves (Fig 2). The study also adhered to the guidelines from the American Veterinary Medicine Association [26] and American Society of Mammalogists for the use of wild mammals in research and education [27].

### Ethics statement

The animal protocol used in this study was approved by the Chilean Animal Health Service (permit number 7034/2017), and by the Scientific Ethics Committee for the Care of Animals and the Environment, Pontificia Universidad Católica de Chile (N°160816007, 07-Nov-2017). All members of the field team were advised and clinically followed for five weeks post-exposure for signs and symptoms of scrub typhus and hantavirus; chemoprophylaxis with doxycycline was not used.

### Analysis of rodent data

A descriptive analysis of the rodent host community and its infestation pattern with ectoparasites was carried out. The percentage of infected individuals with trombiculid mites per species was estimated. Then, we performed a Generalised Lineal Model (GLM) with binomial errors to assess effects of rodent species, and sites on the chigger prevalence using R, version 3.4.1 [28].

### Molecular testing for *Orientia* DNA

Morphologically identical mites from individual rodents were tested as pools of 6–20 individuals. Pools were washed in distilled water and dried in 37°C for 3 hours. Then, mites were disrupted with a freeze-thaw cycle of 30 minutes at -40°C and 30 minutes at 70°C in 100μL distilled water and 100μL lysis buffer (Cat. 04659180001, Roche). Pooled total DNA was automatically extracted by MagNA Pure System (Roche, Pleasanton, California, Roche Molecular Diagnostics), following the manufacturer´s instructions, to a final elution volume of 50 μL [29]. Mite pools were tested by a newly designed quantitative real-time PCR (qPCR) assay targeting the *rrs* gene (16S RNA), which successfully detects all known *Orientia* species including *Candidatus* O. chuto and Chilean *Orientia* isolates [30].

## Results

**Fig 3:**
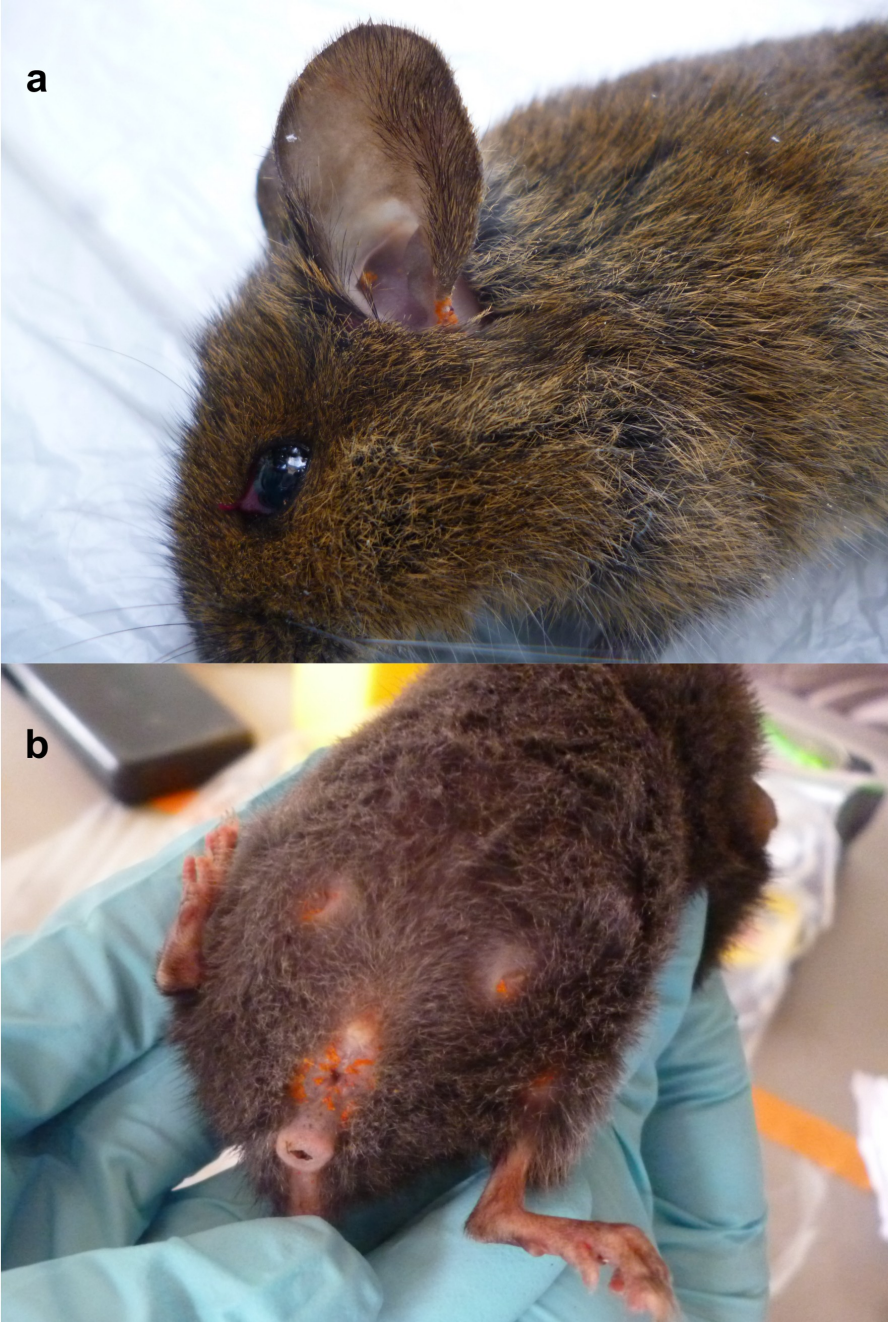
Multiple orange colored trombiculid mites on ear (a) of a *Loxodontomys micropus*, and on the genital region and tits of a *Geoxus valdivianus* (b), captured on Chiloé Island.

Of the captured animals, 244 rodents were included, belonging to seven species, most abundantly *Abrothrix olivacea* (Table 1). Among the trapped animals, 55% (133/244) were infested with trombiculid mites, mainly found in the ears and anogenital region (Fig 3). Other collected ectoparasites included ticks, fleas, and non-trombiculid mites (data not presented). Chigger infestation was observed among all rodent species, with prevalence rates ranging from 77% in *G*. *valdivianus* to 32% in *Irenomys tarsalis*, without significant species-specific differences (GLM, p>0.05). Overall, the most abundant species (*Abrothrix olivacea*) represented 77% (102/133) of all infected animals.

**Table 1:** Infestation with different genera of trombiculid mites and infection of mite pools with *Orientia* species per rodent species in 244 rodents captured between January and February 2018 on Chiloé Island.

Rodent species	Trapped	Infested by trombiculid mites				Mite pools		
	n (%)^1^	Any (%)^2^	*Herpetacarus*	*Quadraseta*	*Paratrombicula*	Total	*Orientia* pos.	*%*
*Abrothrix olivacea*	185 (76)	102 (55)	94	8	8	102	17	16.7
*Abrothrix sanborni*	13 (5)	9 (69)	9	0	0	9	3	33.3
*Geoxus valdivianus*	13 (5)	10 (77)	10	0	0	10	1	10
*Irenomys tarsalis*	25 (10)	8 (32)	7	1	1	8	0	0
*Oligoryzomys longicaudatus*	3 (1)	1 (33)	1	0	0	1	0	0
*Loxodontomys micropus*	3 (1)	2 (67)	2	0	0	2	0	0
*Rattus norvegicus*	2 (1)	1 (50)	1	0	0	1	0	0
Total	244	133 (55)	124	9	9	133	21	15.8

^1^ Percentage of rodent species (among trapped rodent)

^2^ Percentage of infested rodents (among trapped rodents)

Among the collected trombiculids, three morphotypes were observed, which were identified as *Herpetacarus* sp., *Quadraseta* sp., and *Paratrombicula* sp.; details of the detected species will be published elsewhere. *Herpetacarus* sp. was predominate (93% of infested rodents) and occurred on all rodent species and in all sites except Site 3, whereas the other two species co-parasitized on few rodents only in Site 3 (Table 2). The overall prevalence of chiggers in different sites showed significant variations and ranged from 25% to 78% (Table 2). These spatial differences were also presented in a Generalized Lineal Model, demonstrated that it was less likely to find positive rodents in Sites 3 and 5 than in other sites (Table 3).

**Table 2:** Prevalence of infestation with trombiculid mites and infection of mite pools with *Orientia* species per site and rodent species.

Site and host species	Trapped rodents	Mite infested rodents				Mite pools		
		Total		*Herpetacarus*	*Quadraseta*	*Paratrombicula*	Total	*Orientia* pos.	
	n	n	%	n	n	n	n	n	%
**Site 1**	18	12	67	12	0	0	12	0	0
*Abrothrix olivacea*	14	9	64	9	0	0	9	0	0
*Geoxus valdivianus*	4	3	75	3	0	0	3	0	0
**Site 2**	43	31	72	31	0	0	31	6	19.4
*Abrothrix olivacea*	34	23	67	23	0	0	23	5	21.7
*Geoxus valdivianus*	5	5	100	5	0	0	5	1	20.0
*Oligoryzomys longicaudatus*	3	2	67	2	0	0	2	0	0
*Loxodontomys micropus*	1	1	100	1	0	0	1	0	0
**Site 3**	36	9	25	0	9	9	9	0	0
*Abrothrix olivacea*	26	8	31	0	8	8	8	0	0
*Geoxus valdivianus*	1	0	0	0	0	0	0	0	0
*Irenomys tarsalis*	9	1	11	0	1	1	1	0	0
**Site 4**	37	24	65	24	0	0	24	10	41.7
*Abrothrix olivacea*	29	20	69	20	0	0	20	9	45.0
*Abrothrix sanborni*	3	2	67	2	0	0	2	1	50.0
*Geoxus valdivianus*	2	1	50	1	0	0	1	0	0
*Irenomys tarsalis*	1	1	100	1	0	0	1	0	0
*Loxodontomys micropus*	1	0	0	0	0	0	0	0	0
*Rattus norvegicus*	1	0	0	0	0	0	0	0	0
**Site 5**	73	28	38	28	0	0	28	2	7.1
*Abrothrix olivacea*	54	20	37	20	0	0	20	1	5.0
*Abrothrix sanborni*	6	3	50	3	0	0	3	1	33.3
*Geoxus valdivianus*	1	1	100	1	0	0	1	0	0
*Irenomys tarsalis*	10	3	30	3	0	0	3	0	0
*Loxodontomys micropus*	1	0	0	0	0	0	0	0	0
*Rattus norvegicus*	1	1	100	1	0	0	1	0	0
**Site 6**	37	29	78	29	0	0	29	3	10.3
*Abrothrix olivacea*	28	22	79	22	0	0	22	2	9.1
*Abrothrix sanborni*	4	4	100	4	0	0	4	1	25.0
*Irenomys tarsalis*	5	3	60	3	0	0	3	0	0
**Total**	**244**	**133**	**55**	**124**	**9**	**9**	**133**	**21**	**15.8**

**Table 3:** Generalized Lineal Model with binomial error indicating trapping site as a factor for trombiculid infestation in rodents (n = 244) on Chiloé Island.

Sites	OR*	IC95%	p
1	1.00		
2	1.29	0.39–4.22	0.672
3	0.17	0.05–0.57	0.005
4	0.92	0.28–3.03	0.895
5	0.31	0.10–0.92	0.035
6	1.81	0.52–6.35	0.353

*OR = Odd ratio

*Orientia* DNA was detected by a genus-specific qPCR assay in 21 of 133 (15.8%) mite pools (Table 1). All of orientia-positive mite pools belonged to the genus *Herpetacarus* (21 of 124, 16.9%), whereas pools of other mite genera were orientia-negative (Table 1). Three of the most abundant rodent species, *Abrothrix olivacea*, *Abrothrix sanborni*, *Geoxus valdivianus* harbored all *Orientia* positive mites (Table 1). *Orientia*-positive *Herpetacarus* pools were found in four of six sites with prevalence rates ranging from 7.1% to 41.7% (Table 2).

## Discussion

The presented study aimed to explore the trombiculid fauna on Chiloé Island, an endemic area of scrub typhus in southern Chile. Although scrub typhus in Asia-Pacific is transmitted by chiggers, in Chile and other possible newly identified endemic regions, the vector is unknown. Interestingly, a study from 2018 found the first molecular evidence for *Candidatus* Orientia chuto infection of chiggers collected from a rodent in Kenya [31], another region outside the tsutsugamushi triangle, where scrub typhus might occur as suggested by two recent retrospective surveys, detecting antibodies to *Orientia* spp. in 3% to 5% of febrile patients [5, 31]. In the endemic regions in Asia-Pacific, the disease is transmitted by different *Leptotrombidium* species; however, mites of this genus are not endemic in Chile or any other region of the Neotropics [32]. Since the first Chilean scrub typhus patient suffered several terrestrial leech bites prior to his infection, it was speculated that *Orientia* was leech-transmitted [2]. This hypothesis cannot be corroborated by our data. None of the cases diagnosed by our group reported leech bites, at least one had symptoms compatible with trombiculidiasis, and most had nature activities with increased risk of arthropod exposure [3, 33, 34], suggesting that a transmission by chiggers is more likely.

The study was conducted during the typical scrub typhus season in southern Chile, i.e. the summer months of January and February, and locations were chosen according to our previous studies as possible hot spots of *Orientia* transmission [3, 35]. We could demonstrate that rodent-associated chigger mites were present in all those sites. The three detected trombiculid genera have been described before in the Neotropics. *Herpetacarus* was most abundant and parasitized all of the captured rodent species. This genus currently includes four subgenera (*Abonnencia*, *Cricacarus*, *Herpetacarus*, and *Arisocerus*), which are found on reptiles, mammals, and occasionally birds in Africa, Asia, Oceania, and Latin America [36, 37]. Members of this genus have not been detected in Chile, although a closely related genus (*Proschoengastia*) was found in Chile’s far south [14]. The other two morphotypes (*Quadraseta* sp. and *Paratrombicula* sp.) were less prevalent. *Quadraseta*, which comprises 14 species found on rodents and birds [38–45], has not been reported previously in Chile. *Paratrombicula* includes six species isolated from lizards and rodents; two of those (*P*. *chilensis* and *P*. *goffi*) have been described in central Chile, both on lizards [14, 15, 46]. Results of detailed morphological analysis and descriptions of probable new species will be presented elsewhere.

Rodents are a main reservoir of chiggers and important determinant of the distribution of scrub typhus in endemic regions in Asia [6]. In our study, *A*. *olivacea* was the most abundant rodent species and also represented the highest (absolute) number of infested rodents. This species is a typical inhabitant of Valdivian temperate forests; showing a large numerical response during bamboo blossom (*Chusquea* spp.) [47–49]. Other less abundant host species were infested in similar rates, indicating low host-specificity of trombiculids. This is in accordance with studies from Asia-Pacific, where feeding on small mammals was non-specific and depended on host species abundance in the community [1, 7, 8].

Although chiggers were detected on rodents in all six surveyed areas, the prevalence rates differed geographically. Variations in the distribution of trombiculids are a known phenomenon; in fact, within suitable habitats, the mites usually have a patchy distribution, forming so called “mite islands” [9, 50]. Our findings might indicate a high prevalence of chiggers on Chiloé Island, although the selection of sites as probable “hot spots” of exposure to *Orientia* spp. could be a bias towards overestimation. The infestation rates reported in this study are comparable to those reported in the Asia-Pacific region where prevalence rates on small mammals ranged from 45% to 95% [7, 8, 51, 52].

Trombiculid mites live in moist soil covered with vegetation and are mostly found in grassy and weedy areas [eg. 50]. Optimal living conditions depend on various factors such as air humidity, soil composition, temperature, and light intensity. Habitat fragmentation seems to affect mite survival by modifying their ecological niche [53]. Recently, a large-scale research found higher infestation (prevalence, mean abundance, and intensity) with vector mites on small mammals in areas with lower biodiversity compared to those with higher biodiversity [54]. As documented in various studies, forest fragmentation on Chiloé Island reduces the biodiversity of small mammals, non-raptorial birds, and the predator assemblage and increases the abundance of generalist species [55–58]. In a similar manner, fragmentation might affect rodent populations and their associated trombiculid ectoparasites.

This first demonstration of rodent-associated chiggers in probable hot spots of scrub typhus suggests that chiggers might serve as vectors of this infection in Chile. The study detected high chigger prevalence in the summer season, during which up to now all cases of scrub typhus in Chiloé have occurred [59]. The hypothesis was further supported by the detection of *Orientia* DNA in 15.8% of mites, all of which belonged to the *Herpetacarus* genus. Final proof, however, of the vector competence of the detected trombiculid mites in this new endemic region requires further studies. To understand the risk of human exposure to trombiculid mites, further investigations are necessary, which should include environmental, anthropogenic, and climatic variables influencing the epidemiology of these potential vectors in southern Chile.

### Conclusions

Our study firstly documented the presence of rodent-associated trombiculid mites infected with *Orientia* sp. on Chiloé Island, a region endemic for scrub typhus in South America. Three different mite genera were identified on the rodents with the genus *Herpetacarus* the most abundant and the only infected with *Orientia* sp. Overall, we detected a high rate of *Orientia*-infected chigger infestation independent of host species, but with significant spatial variations.

## References

[1] Luce-Fedrow A, Lehman ML, Kelly DJ, Mullins K, Maina AN, Stewart RL, et al A Review of Scrub Typhus (*Orientia tsutsugamushi* and Related Organisms): Then, Now, and Tomorrow. Tropical medicine and infectious disease. 2018;3 10.3390/tropicalmed3010008 30274407 PMC6136631

[2] Balcells ME, Rabagliati R, Garcia P, Poggi H, Oddo D, Concha M, et al Endemic scrub typhus-like illness, Chile. Emerg Infect Dis. 2011;17(9):1659–63. 10.3201/eid1709.100960 21888791 PMC3322051

[3] Weitzel T, Dittrich S, Lopez J, Phuklia W, Martinez-Valdebenito C, Velasquez K, et al Endemic Scrub Typhus in South America. N Engl J Med. 2016;375(10):954–61. 10.1056/NEJMoa1603657 . 27602667

[4] Izzard L, Fuller A, Blacksell SD, Paris DH, Richards AL, Aukkanit N, et al Isolation of a novel Orientia species (O. chuto sp. nov.) from a patient infected in Dubai. J Clin Microbiol. 2010;48(12):4404–9. 10.1128/JCM.01526-10 20926708 PMC3008486

[5] Xu G, Walker DH, Jupiter D, Melby PC, Arcari CM. A review of the global epidemiology of scrub typhus. PLoS Negl Trop Dis. 2017;11(11):e0006062 10.1371/journal.pntd.0006062 29099844 PMC5687757

[6] Coleman RE, Monkanna T, Linthicum KJ, Strickman DA, Frances SP, Tanskul P, et al Occurrence of *Orientia tsutsugamushi* in small mammals from Thailand. Am J Trop Med Hyg. 2003;69(5):519–24. . 14695089

[7] Kuo C-C, Lee P-L, Chen C-H, Wang H-C. Surveillance of potential hosts and vectors of scrub typhus in Taiwan. Parasite Vector. 2015;8:611 10.1186/s13071-015-1221-7 PMC4666075. 26626287 PMC4666075

[8] Kuo CC, Huang CL, Wang HC. Identification of potential hosts and vectors of scrub typhus and tick-borne spotted fever group rickettsiae in eastern Taiwan. Med Vet Entomol. 2011;25(2):169–77. 10.1111/j.1365-2915.2010.00941.x . 21223345

[9] Traub R, Wisseman CL Jr. The ecology of chigger-borne rickettsiosis (scrub typhus). J Med Entomol. 1974;11(3):237–303. 10.1093/jmedent/11.3.237 . 4212400

[10] Santibáñez P, Palomar A, Portillo A, Santibáñez S, Oteo JA. The role of chiggers as human pathogens In: Samie A, editor. An Overview of Tropical Diseases: Intech Open Limited, LDN; 2015 p. 173–202.

[11] Tilak R, Kunwar R, Wankhade UB, Tilak VW. Emergence of *Schoengastiella ligula* as the vector of scrub typhus outbreak in Darjeeling: has *Leptotrombidium deliense* been replaced? Indian J Public Health. 2011;55(2):92–9. 10.4103/0019-557X.85239 . 21941043

[12] Lee HW, Cho PY, Moon SU, Na BK, Kang YJ, Sohn Y, et al Current situation of scrub typhus in South Korea from 2001–2013. Parasit Vectors. 2015;8:238 10.1186/s13071-015-0858-6 25928653 PMC4416255

[13] Wei Y, Huang Y, Luo L, Xiao X, Liu L, Yang Z. Rapid increase of scrub typhus: an epidemiology and spatial-temporal cluster analysis in Guangzhou City, Southern China, 2006–2012. PLoS One. 2014;9(7):e101976 10.1371/journal.pone.0101976 25006820 PMC4090214

[14] Stekolnikov AA, Gonzalez-Acuna D. A review of Chilean chiggers (Acari: Trombiculidae), with the description of a new genus and ten new species. Zootaxa. 2015;3964(1):1–43. 10.11646/zootaxa.3964.1.1 . 26249418

[15] Silva de la Fuente MC, Casanueva ME, Salas LM, Gonzalez-Acuna D. A new genus and species of chigger mite (Trombidiformes: Trombiculidae) from *Loxodontomys pikumche* (Rodentia: Cricetidae) in Chile. Zootaxa. 2016;4092(3):426–30. 10.11646/zootaxa.4092.3.8 . 27394465

[16] Espinoza-Carniglia M, Pérez-Leiva A, Silva de la Fuente MC, Victoriano-Sepúlveda P, Moreno-Salas L. Abundance and distribution of parasitic mites (*Eutrombicula araucanensis* and *Pterygosoma* sp.) on lizards (*Liolaemus pictus*) of central Chile. Rev Mex Biodivers. 2016;87:101–8.

[17] Sarricolea P, Herrera-Ossandon MJ, Meseguer-Ruiz O. Climatic regionalisation of continental Chile. J Maps 2017;13:66–73.

[18] Jaña-Prado R, Celis-Diez JL, Gutierrez AG, Cornelius C, Armes to JJ. Diversidad en los bosques fragmentados de Chiloe: ¿son todos los fragmentos iguales? In: Grez AA, Simonetti JA, Bustamante RO, editors. Biodiversidad en ambientes fragmentados de Chile: patrones y procesos a diferentes escalas. Santiago, Chile: Editorial Universitaria; 2006 p. 159–90.

[19] Aravena JC, Carmona MR, Pérez CA, Armesto JJ. Changes in tree species richness, stand structure and soil properties in a successional chronosequence in northern Chiloé Island, Chile. Rev Chil Hist Nat. 2002;75:339–60.

[20] Iriarte A. Mamíferos de Chile. Barcelona, España: Lynx; 2008. 420 p.

[21] Walter DE, Krantz GW. Chapter 7: Collecting, Rearing, and Preparing Specimens In: Krantz GW, Walter DE, editors. A Manual of Acarology. 3rd ed Lubbock, Texas: Texas Tech University Press; 2009 p. 83–96.

[22] Brennan JM, Goff ML. Keys to the genera of chiggers of the Western Hemisphere (Acarina: Trombiculidae). J Parasitol. 1977;63:554–66. 68115

[23] Medina RA, Torres-Perez F, Galeno H, Navarrete M, Vial PA, Palma RE, et al Ecology, genetic diversity, and phylogeographic structure of andes virus in humans and rodents in Chile. J Virol. 2009;83(6):2446–59. 10.1128/JVI.01057-08 19116256 PMC2648280

[24] Mills JN, Yates TL, Childs JE, Parmenter RR, Ksiazek TG, Rollin PE, et al Guidelines for working with rodents potentially infected with Hantavirus. J Mammal. 1995;76(3):716–22. ISI:A1995RQ81100005.

[25] Kelt DA, Hafner MS, The American Society of Mammalogists' ad hoc Committee for Guidelines on Handling Rodents in the F. Updated guidelines for protection of mammalogists and wildlife researchers from hantavirus pulmonary syndrome (HPS). J Mammal. 2010;91(6):1524–7. 10.1644/10-MAMM-A-306.1

[26] Leary S, Underwood W, Anthony R, Cartner S. AVMA Guidelines for the Euthanasia of Animals: 2013 Edition2013.

[27] Sikes RS, Animal C, Use Committee of the American Society of Mammalogists. 2016 Guidelines of the American Society of Mammalogists for the use of wild mammals in research and education. J Mammal. 2016;97(3):663–88. 10.1093/jmammal/gyw078 29692469 PMC5909806

[28] R-Development-Core-Team. R: A Language and Environment for Statistical Computing. Vienna, Austria: the R Foundation for Statistical Computing Available online at http://www.R-project.org/. 2016.

[29] Sahu SK, Thangaraj M, Kathiresan K. DNA Extraction Protocol for Plants with High Levels of Secondary Metabolites and Polysaccharides without Using Liquid Nitrogen and Phenol. ISRN molecular biology. 2012;2012:205049 10.5402/2012/205049 27335662 PMC4890884

[30] Jiang J, Martínez-Valdebenito C, Weitzel T, Abarca K, Richards AL. Development of an *Orientia* genus-specific quantitative real-time PCR assay and the detection of *Orientia* species in DNA preparations from *O. tsutsugamushi*, *Candidatus* Orientia chuto, and *Orientia* species from Chile. Abstracts of the 29th Meeting of the American Society for Rickettsiology; Milwaukee, USA; 2018 Jun 16–19, Abstract no 46.

[31] Masakhwe C, Linsuwanon P, Kimita G, Mutai B, Leepitakrat S, Yalwala S, et al Identification and characterization of *Orientia chuto* in trombiculid chigger mites collected from wild rodents in Kenya. J Clin Microbiol. 2018;56(12). 10.1128/JCM.01124-18 30282787 PMC6258837

[32] Stekolnikov AA. Leptotrombidium (Acari: Trombiculidae) of the World. Zootaxa. 2013;3728:1–173. 10.11646/zootaxa.3728.1.1 . 26079025

[33] Weitzel T, Martínez-Valdebenito C, Acosta-Jamett G, Jiang J, Richards AL, Abarca K. Scrub typhus in continental Chile, 2016–2018. Emerging Infectious Diseases. 2019;25(6):1214 10.3201/eid2506.181860. 30835200 PMC6537721

[34] Weitzel T, Acosta-Jamett G, Martinez-Valdebenito C, Richards AL, Grobusch MP, Abarca K. Scrub typhus risk in travelers to southern Chile. Travel Med Infect Dis. 2019;29:78–79. 10.1016/j.tmaid.2019.01.004 . 30639781

[35] Weitzel T, Jiang J, Acosta-Jamett G, Martinez-Valdebenito C, Lopez J, Richards AL, et al Canine seroprevalence to Orientia species in southern Chile: A cross-sectional survey on the Chiloe Island. PLoS One. 2018;13(7):e0200362 10.1371/journal.pone.0200362 . 29979764 PMC6034878

[36] Jacinavicius FC, Bassini-Silva R., Welbourn C., Ochoa R. & Barros-Battesti D.M. Synonymy of the genus *Arisocerus* Brennan, 1970 with the genus *Herpetacarus* Vercammen-Grandjean, 1960 (Trombidiformes: Trombiculidae). Systematic & Applied Acarology. 2019;24:1138–49.

[37] Vercammen-Grandjean PH. Revision of the genus *Herpetacarus* Vercammen-Grandjean, 1960 (Trombiculidae, Acarina). Acarologia. 1966;8:631–74.

[38] Goff ML, Gettinger D. Two New Species of Schoengastiin e Chiggers (Acari: Trombiculidae) from Brazil and Rediagnosis of Arisocerus Brennan, 1970. Journal of Medical Entomology. 1989;26(6): 554–8. 10.1093/jmedent/26.6.554 2585450

[39] Goff ML, Gettinger D. New genus and six new species of chiggers (Acari: Trombiculidae and Leeuwenhoekiidae) collected from small mammals in Argentina. Journal of Medical Entomology. 1995;32:439–48. 10.1093/jmedent/32.4.439 7650704

[40] Brennan JM, Jones EK. Four New Chiggers from Argentina (Acarina: Trombiculidae). J Parasitol. 1964;50(5):698–702. 14215495

[41] Brennan JM, Jones EK. Five new species of chiggers from South America (Acarina: Trombiculidae). Journal of Medical Entomology. 1964;1(3):307–10. 14222872 10.1093/jmedent/1.3.307

[42] Goff ML, Brennan JM. A new monotypic genus of chiggers and four new species of Quadraseta from Venezuela (Acari: Trombiculidae). Great Basin Naturalist. 1977;37(4):501–9.

[43] Goff ML, Whitaker JOJ. A small collection of chiggers (Acari: Trombiculidae) from mammals collected in Paraguay. Journal of Medical Entomology. 1984;21(3):327–35. 10.1093/jmedent/21.3.327 6748009

[44] Yunker CE, Brennan JM. Four new chiggers (Acarina: Trombiculidae) from rodents of the epidemic area of bolivian hemorrhagic fever. Journal of Medical Entomology. 1964;1:192–5.

[45] Brennan JM, Jones EK. New genera and species of chiggers from Panama (Acarina: Trombiculidae). J Parasitol. 1961;67:105–24.

[46] Stekolnikov AA, González-Acuña D. A revision of the chigger mite genus Paratrombicula Goff & Whitaker, 1984 (Acari: Trombiculidae), with the description of two new species. Syst Parasitol. 2012;83:105–15. 10.1007/s11230-012-9373-8 22983798

[47] Gonzalez LA, Murúa R, Jofre C. Habitat utilization of two muroid species in relation to population outbreaks in southern temperate forests of Chile. Rev Chil Hist Nat. 2000;73:489–95.

[48] Murúa R, González LA. Microhabitat selection in two Chilean cricetid rodents. Oecologia. 1982;52(1):12–5. 10.1007/BF00349005 28310102

[49] Spotorno AE, Palma RE, Valladares JP. Biología de roedores reservorios de hantavirus en Chile. Rev Chilena Infectol. 2000;17:197–210.

[50] Peng PY, Guo XG, Ren TG, Song WY, Dong WG, Fan R. Species diversity of ectoparasitic chigger mites (Acari: Prostigmata) on small mammals in Yunnan Province, China. Parasitol Res. 2016;115(9):3605–18. 10.1007/s00436-016-5127-x . 27212464

[51] Ree HI, Lee IY, Jeon SH, Yoshida Y. Geographical distribution of vectors and sero-strains of tsutsugamushi disease at mid-south inland of Korea. Korean J Parasitol. 1997;35(3):171–9. 10.3347/kjp.1997.35.3.171 . 9335182

[52] Lin P-R, Tsai H-P, Weng M-H, Lin H-C, Chen K-C, Kuo M-D, et al Field assessment of Orientia tsutsugamushi infection in small mammals and its association with the occurrence of human scrub typhus in Taiwan. Acta Tropica. 2014;131:117–23. 10.1016/j.actatropica.2013.11.029 24361181

[53] Espinoza-Carniglia M, Silva De La Fuente MC, Pérez A, Victoriano PF, Moreno-Salas L. Fragmented host distribution and trombiculid parasitic load: *Eutrombicula araucanensis* and *Liolaemus pictus* in Chile. Acarologia. 2015;55:209–17.

[54] Peng PY, Guo XG, Jin DC, Dong WG, Qian TJ, Qin F, et al Landscapes with different biodiversity influence distribution of small mammals and their ectoparasitic chigger mites: A comparative study from southwest China. PLoS One. 2018;13(1):e0189987 10.1371/journal.pone.0189987 29364908 PMC5783360

[55] Willson MF, Sieving KE, De Santo TL. Aves del bosque de Chiloé: diversidad, amenazas y estrategias de conservación In: Smith-Ramirez C, Armesto JJ, Valdovinos C, editors. Historia, biodiversidad y ecología de los bosques costeros de Chile Santiago, Chile: Editorial Universitaria; 2005 p. 468–76.

[56] Armesto JJ, Willson MF, Diaz I, Reid S. Ecologia del paisaje rural de la isla de Chiloe: diversidad de aves en fragmentos de bosque nativo In: Smith-Ramirez C, Armesto JJ, Valdovinos C, editors. Historia, biodiversidad y ecología de los bosques costeros de Chile Santiago, Chile: Editorial Universitaria; 2005 p. 585–99.

[57] Farias AA, Jaksic FM. Low functional richness and redundancy of a predator assemblage in native forest fragments of Chiloe Island, Chile. Journal of Animal Ecology. 2011;80:809–17. 10.1111/j.1365-2656.2011.01824.x 21361929

[58] Jimenez JE. Ecology of a coastal population of the critically endangered Darwin's fox (*Pseudalopex fulvipes*) on Chiloe Island, southern Chile. J Zool. 2007;271(1):63–77. 10.1111/j.1469-7998.2006.00218.x WOS:000243240200009.

[59] Abarca K, Weitzel T, Martinez-Valdebenito C, Acosta-Jamett G. [Scrub typhus, an emerging infectious disease in Chile]. Rev Chilena Infectol. 2018;35(6):696–9. 10.4067/S0716-10182018000600696 . 31095191

